# Clinical Manifestations, Antimicrobial Drug Susceptibility Patterns, and Outcomes in Melioidosis Cases, India

**DOI:** 10.3201/eid2502.170745

**Published:** 2019-02

**Authors:** Maria Koshy, Manjeera Jagannati, Ravikar Ralph, Punitha Victor, Thambu David, Sowmya Sathyendra, Balaji Veeraraghavan, George M. Varghese

**Affiliations:** Christian Medical College, Vellore, India

**Keywords:** melioidosis, Burkholderia pseudomallei, clinical manifestations, antimicrobial drug susceptibility, outcomes, bacteria, India

## Abstract

We studied the clinical manifestations and outcomes of 114 patients with culture-confirmed melioidosis treated at a tertiary hospital in southern India. Diabetes mellitus is the main risk factor, and chronic melioidosis mimicking tuberculosis was more common than acute disease. Septicemia and respiratory involvement were associated with poor outcomes.

Melioidosis is a tropical infection caused by the environmental bacterium *Burkholderia pseudomallei* (*1*). The clinical spectrum of melioidosis ranges from the acute septicemic form, which is often fulminant, to chronic disease, which mimics other common infections like tuberculosis (*2*). Melioidosis is endemic to southeast Asia and northern Australia; seasonal peaks occur during monsoons (*3*). Infection is acquired by inhalation, inoculation, and ingestion (*4*).

Melioidosis is often difficult to diagnose because of diverse clinical manifestations, low index of suspicion, and poor availability of good laboratory facilities. Hence, the disease remains underrecognized even in endemic countries. Recent studies have shown that India is at high risk for a surge of cases of melioidosis (*5*). Our study was undertaken to delineate the clinical manifestations, antimicrobial drug susceptibility patterns, and outcomes of melioidosis cases in India.

## The Study

This retrospective study included 114 adult patients with culture-confirmed melioidosis treated at a tertiary care teaching hospital in southern India during January 2008–December 2014. We collected data regarding patient demographics, clinical characteristics, laboratory results, radiologic parameters, and antimicrobial drug susceptibility from medical records. We classified patients on the basis of duration of symptoms: acute (<2 months) or chronic (>2 months) (*6*). We defined multifocal disease as involvement of >2 organs and disseminated disease as involvement of 1 organ plus bacteremia (*7*). We assessed severity of illness (based on sequential organ failure assessment [SOFA] scores), complications, and patient outcomes (*8*). We used the BacT ALERT (bioMérieux, https://www.biomerieux.com) automated system for blood culture. We plated pus and tissue on blood, chocolate, and MacConkey agar and identified *B. pseudomallei* according to standard methods and performed antimicrobial drug susceptibility testing according to Clinical and Laboratory Standards Institute standards using E-test for trimethoprim/sulfamethoxazole. We summarized descriptive data and analyzed the association of patient parameters with outcomes by using logistic regression. We considered a 2-sided p value of <0.05 statistically significant. We analyzed the data using SPSS Statistics for Windows version 20.0 (IBM, https://www.ibm.com).

**Figure 1:**
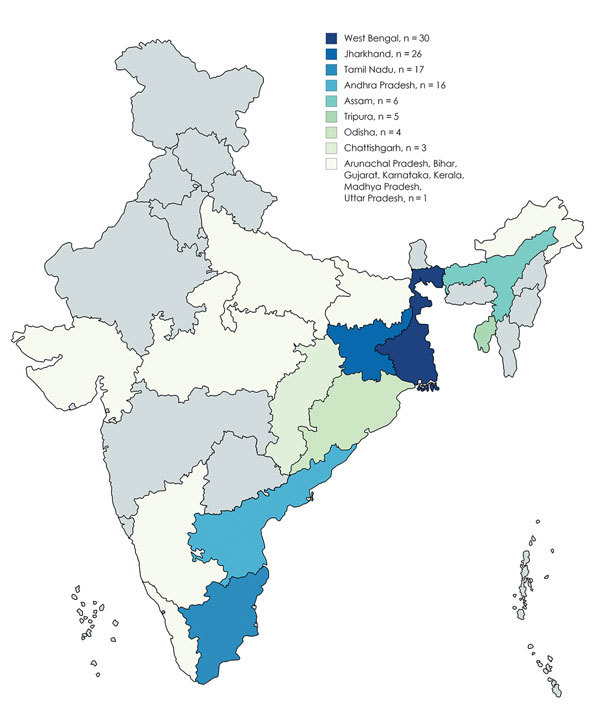
Distribution of patients with melioidosis, by state, India, 2008–2014.

Patients were predominantly male (92%); mean age was 45.6 years (33.5–57.7 years). The patients came from 15 states in India; most were from West Bengal (26.3%), Jharkhand (22.8%), and Tamil Nadu (14.9%) (Figure 1). The common risk factors identified were diabetes mellitus (81.6%), harmful ethanol consumption (14%), and chronic kidney disease (3.5%). Among patients with diabetes, glycemic control was poor (mean hemoglobin A1c 9.7% [7.1%–12.3%]). Other risk factors included sickle cell disease, thalassemia, glucocorticoid use, and liver disease (Table 1). Patients with acute disease more commonly sought care (80.5%, n = 33) during the cooler months of August–February.

**Table 1:** Patient characteristics and outcomes of patients with melioidosis, southern India, 2008–2014*

Characteristic	All patients, N = 114	Acute melioidosis, n = 41	Chronic melioidosis, n = 73
Age, y, mean + SD	45.6 + 12.1	46 + 12.6	45.3 + 11.9
Sex, no., M/F	105/9	38/3	67/6
Comorbid conditions, no. (%)			
Diabetes mellitus	93 (81.6)	36 (87.8)	57 (78.1)
Harmful ethanol use	16 (14)	10 (24.4)	6 (8.5)
Chronic kidney disease	4 (3.5)	2 (4.9)	2 (2.8)
Sickle cell disease	3 (2.6)	0	3 (4.2)
No risk factors	15 (13.2)	3 (7.3)	12 (16.4)
Duration of symptoms, d, median (IQR)	60 (30–90)	20 (7–30)	90 (60–210)
Clinical symptoms, no. (%)			
Fever	105 (97.2)	41 (100)	64 (95.5)
Cough	17 (15.5)	9 (21.9)	8 (11.7)
Abdominal pain	38 (34.8)	10 (24.3)	28 (41.1)
Joint pain	27 (24.7)	10 (24.3)	17 (25)
Breathlessness	16 (14.6)	11 (26.8)	5 (7.3)
Clinical signs, no. (%)			
Tachypnea, reference >24/min	24 (21.2)	14 (34.1)	10 (13.9)
Tachycardia, reference >100/min	33 (29.2)	20 (48.8)	13 (18.1)
Hypotension, reference <90/60 mm Hg	11 (9.7)	6 (14.6)	5 (6.9)
Laboratory parameters			
Hemoglobin, g/dL, mean + SD	10.2 + 2.3	11.3 + 2.5	9.6 + 2
Lymphocyte count, × 10^9^ cells/L, median (IQR)	10.5 (7.7–15.4)	14.6 (11.1–19.4)	9 (6.8–12.2)
Platelet count, x 10^9^/L, median (IQR)	165 (107–272)	177 (115–264)	158 (102–280)
Total bilirubin, mg/dl, median (IQR)	0.7 (0.5–1.4)	1.2 (0.7–1.8)	0.6 (0.4–0.9)
Total protein, g/dL, mean + SD	7.2 + 1	6.7 + 0.8	7.5 + 1
Albumin, g/dL, mean + SD	2.9 + 0.8	2.6 + 0.7	3.1 + 0.7
AST, IU/mL, median (IQR)	42 (25–85)	48 (26–128)	36 (24–83)
ALT, IU/mL, median (IQR)	29 (15–60)	28 (13–99)	30 (15–56)
ALP, IU/mL, median (IQR)	148 (85–249)	157 (102–239)	137 (83–276)
Serum creatinine, mg/dL, median (IQR)	1 (0.8–1.3)	1.1 (0.8–1.3)	1 (0.8–1.2)
Hemoglobin A1C, %, mean + SD	9.7 + 2.6	11.1 + 2.9	8.9 + 2
CRP, mg/L, mean + SD	96.6 + 64.3	152.7 + 57.2	77.4 + 55.2
Organ involvement			
Lung	28 (24.5)	16 (39)	12 (16.4)
Bacteremia	63 (55.2)	33 (80.4)	30 (41)
Spleen	49 (42.9)	12 (29.2)	37 (50.6)
Liver	25 (21.9)	9 (21.9)	16 (21.9)
Genitourinary	16 (14)	2 (4.8)	14 (19.1)
Septic arthritis	22 (19.2)	9 (21.9)	13 (17.8)
Osteomyelitis	12 (10.5)	3 (7.3)	9 (12.3)
Skin and subcutaneous tissue	15 (13.1)	7 (17)	8 (10.9)
Parotid	2 (1.7)	2 (4.8)	0
Central nervous system	3 (2.6)	2 (4.8)	1 (1.3)
Sequential organ failure assessment score, median (IQR)	2 (1–4)	3 (1–6)	1 (0–3)
ICU admission, no. (%)	25 (21.9)	15 (36.6)	10 (13.7)
Mechanical ventilation, no. (%)	21 (18.4)	13 (31.7)	8 (11)
Duration of hospitalization, days, median (IQR)	16.5 (9–24)	18 (8.5–30.5)	16 (10–21.5)
Case-fatality rate, no. (%)	17 (14.9)	7 (17.1)	10 (13.7)

*ALP, alkaline phosphatase; ALT, alanine aminotransferase; AST, aspartate aminotransferase; CRP, C-reactive protein; ICU, intensive care unit; IQR, interquartile range.

Chronic melioidosis was more common than acute melioidosis (64% vs. 36%) in this patient cohort. Duration of symptoms was 2 days to 5 years (median 60 days; interquartile range [IQR] 30–90 days). Fever was the most consistent symptom (97.2%). Although other studies have reported lung involvement to be most common (*9*), we most frequently observed splenic (42.9%) and musculoskeletal (37.7%) disease (Table 1). Chronic melioidosis was associated with splenic and genitourinary involvement. Prostatic abscess was the most common genitourinary disease (87.5%, n = 12). Multifocal disease was evident in 89 (78%) patients. Two patients had tuberculosis–melioidosis co-infection and improved with appropriate therapy for both diseases. Chest radiograph abnormalities included bilateral fluffy opacities (16.7%, n = 19), focal infiltrates (6.1%, n = 7), and effusions (6.1%, n = 7).

Patients with acute melioidosis frequently had respiratory involvement and bacteremia. Septic arthritis was common in acute disease and usually involved a single large joint. One patient had evidence of multifocal arthritis. Three patients had central nervous system involvement. Five patients had bacteremia without evidence of deep-seated abscesses. We also observed unusual presentations, such as pancreatitis and pericardial involvement. In 11 (15.1%) patients with chronic melioidosis, the condition worsened because of bacteremia and systemic inflammatory response syndrome. This phenomenon occurred especially in elderly patients and patients with diabetes and was associated with higher case-fatality rates.

A total of 63 (55.2%) patients had bacteremia, and 25 (21.9%) patients required admission to intensive care. Seventy-six (66.6%) patients required a diagnostic or therapeutic interventional procedure. Antimicrobial drug susceptibility tests revealed 100% sensitivity to carbapenems and ceftazidime. Resistance to trimethoprim/sulfamethoxazole was noted in 5.9% and resistance to doxycycline in 2.6% of cases.

Patients received intensive therapy with meropenem or ceftazidime for a median duration of 16 days (IQR 14–28 days), followed by eradication therapy with trimethoprim/sulfamethoxazole with or without doxycycline for a median of 90 days (IQR 90–150 days). Most patients were treated successfully with these regimens. Follow-up data were available for 57.9% patients for a period of 3 months to 1 year. Four (3.5%) patients had disease recurrence 2–7 years after completion of therapy, of whom 1 received inadequate therapy. A previous study highlighted that multifocal disease, bacteremia, and duration of therapy correlate with risk for relapse (*6*).

**Figure 2:**
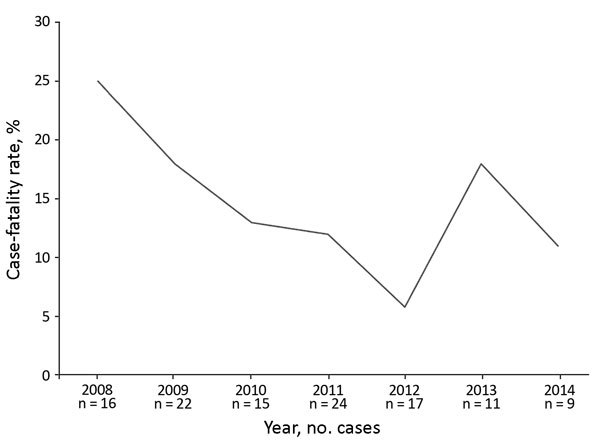
Trend in melioidosis case-fatality rates, southern India, 2008–2014.

The overall case-fatality rate was 14.9% (n = 17) and was higher in patients with acute disease (Table 2). On multivariate analysis of predictors of mortality risk, respiratory involvement (odds ratio [OR] 4.61 [95% CI 1.57–13.55]; p = 0.005) and bacteremia (OR 17.02 [95% CI 2.17–133.43]; p = 0.007) were independent predictors of mortality, along with admission SOFA scores (OR 1.4 [95% CI 1.1–1.7]; p<0.001) and hypoalbuminemia (OR 4.02 [95% CI 1.21–13.33]; p = 0.02). We noticed a decline in case-fatality rate from 2008 (25%) to 2014 (11%) (Figure 2).

**Table 2:** Bivariate analysis of predictors of mortality from melioidosis, southern India, 2008–2014

Predictors of mortality	Alive, n = 97	Dead, n = 17	Odds ratio (95% CI)	p value
Diabetes mellitus	79	14	0.94 (0.23–3.62)	0.92
Harmful ethanol use	14	2	0.90 (0.18–4.43)	0.89
Chronic kidney disease	3	1	0.45 (0.04–4.65)	0.5
Tachypnea, reference >24/min	12	12	16.8 (5.03–53.11)	<0.001
Tachycardia, reference >100/min	22	11	6.16 (2.04–18.57)	0.001
Hypotension, reference <90/60 mmHg	4	7	16.10 (4.00–64.70)	<0.001
Sequential organ failure assessment score	1 (0–3)	4 (2–8)	1.4 (1.1–1.7)	<0.001
Bacteremia	47	16	17.02 (2.17–133.43)	0.007
Respiratory involvement	19	9	4.61 (1.57–13.55)	0.005
Hypoalbuminemia, reference <3 g/dL	38	13	4.02 (1.21–13.33)	0.02
Intensive care unit admission	12	13	23.02 (6.44–82.24)	<0.001
Mechanical ventilation	8	13	36.15 (9.52–137.23)	<0.001

## Conclusions

This study highlights the emergence of melioidosis as a major problem in India. Two thirds of our patient population were from the eastern and northeastern parts of India, which might reflect a referral bias. Infection frequently affected the middle-aged, male working population, highlighting possible occupational exposure. The association of acute melioidosis with the wet season in certain regions concurs with other studies that have shown a correlation between rainfall intensity and disease (*10*). The higher proportion of patients with diabetes (82.3%), compared to other studies (39%–60%), might reflect the emerging diabetes epidemic in India (*4*,*11*).

The predominance of chronic melioidosis is a finding not in keeping with other studies (*1*,*4*,*10*) and might be attributed in part to referral bias and referral of undiagnosed cases from relatively dry areas of the country. Patients with acute disease tended to reside near our hospital. The occurrence of an acute worsening in patients with chronic disease was associated with a poor outcome. The observation of tuberculosis–melioidosis co-infection is not unusual given that the risk factors and interferon-mediated host response is common to both conditions (*12*).

Most patients were treated successfully with intensive therapy followed by eradicative therapy. The case-fatality rate associated with melioidosis was 14.9%, with a decreasing trend over the study period. A study previously conducted from this hospital reported a case-fatality rate of 17% (*7*), which is lower than the case-fatality rate in Thailand (≈40%) (*13*). We found that bacteremia, respiratory involvement, hypoalbuminemia, and high SOFA scores were associated with poor outcomes.

We found that 5.9% of *B. pseudomallei* isolates were resistant to trimethoprim/sulfamethoxazole. This percentage might have been a slight overestimate because of the change in antimicrobial drug susceptibility testing methods during the study period. Although a previously reported trial showed noninferiority of trimethoprim/sulfamethoxazole to trimethoprim/sulfamethoxazole plus doxycycline, eradication therapy in our clinical setting should be based on antimicrobial drug susceptibility reports (*14*).

Melioidosis warrants attention from clinicians and public health officials in India. Specific organ involvement can alert clinicians to acute or chronic disease, which might be useful in management of patients in a resource-limited setting. Because of the substantial mortality associated with melioidosis, a high index of suspicion and early initiation of therapy are essential.
